# Combination of Comfrey Root Extract Plus Methyl Nicotinate in Patients with Conditions of Acute Upper or Low Back Pain: A Multicentre Randomised Controlled Trial

**DOI:** 10.1002/ptr.4790

**Published:** 2012-08-08

**Authors:** Helmut Pabst, Axel Schaefer, Christiane Staiger, Marc Junker-Samek, Hans-Georg Predel

**Affiliations:** 1 Independent PhysicianGilching, Germany; 2 Independent PhysicianEssen, Germany; 3 Merck Selbstmedikation GmbHDarmstadt, Germany; 4 German Sport University, Institut für Kreislaufforschung und SportmedizinCologne, Germany

**Keywords:** comfrey, methyl nicotinate, acute back pain, RCT

## Abstract

This randomised, multicentre, double-blind, three-arm, placebo-controlled trial compared a topical combination of 35% comfrey root extract plus 1.2% methyl nicotinate versus a single preparation of methyl nicotinate or placebo cream for relief of acute upper or low back pain. 379 patients were randomly assigned to three groups (combination, *n* = 163; methyl nicotinate, *n* = 164; placebo, *n* = 52). They applied a 12 cm layer of cream three times daily for 5 days. The primary efficacy variable was the area under the curve (AUC) of the visual analogue scale (VAS) on active standardised movement values at visits 1 to 4. Secondary measures included back pain at rest, pressure algometry, consumption of analgesic medication, functional impairment measured with Oswestry Disability Index, and global assessment of response. The AUC of the VAS on active standardised movement was markedly smaller in the combination treatment group than in the methyl nicotinate and in the placebo group (ANOVA: *p* < 0.0001). The combination demonstrated superiority to the two other treatment arms, while methyl nicotinate displayed a considerable effect as well. Copyright © 2012 John Wiley & Sons, Ltd.

Acute back pain of the upper or low back is a widespread condition that impairs quality of life and functional movement in a large number of people (McCarberg, 2010; Balagué *et al*., 2012). However, patients seek for pain relief and treat their pain symptoms often in self-medication. There are many potential causes for acute back pain, and precise causation is difficult to determine. As a consequence, the treatment of acute back pain is a complex issue. Recommended topical and systemic pharmacologic treatments for acute low back pain include application of superficial heat, acetaminophen, nonsteroidal anti-inflammatory drugs (NSAIDs), skeletal muscle relaxants/benzodiazepines, and opioids including tramadol (McCarberg, 2010).

The topical pharmacotherapeutic approach has generally included hyperaemising topical drugs such as nicotinates with the intention to soften and relax the contracted muscle area, thereby indirectly alleviating the pain caused by this contraction. In addition to heat or hyperaemia, the treatment strategy has recently been augmented by a direct anti-inflammatory topical approach – for instance with diclofenac.

Topical treatments with anti-inflammatory and analgesic properties provide an interesting alternative, taking into account the potential adverse effects of oral therapies (e.g. gastrointestinal and renal side effects of NSAIDs and analgesics). Extracts of comfrey in mono preparations and in combination with methyl nicotinate have a long tradition of use as topical treatment (Englert *et al*., 2005; Staiger, 2005; Staiger, 2007; European Scientific Cooperative on Phytotherapy (ESCOP), 2009; Staiger, 2012) In clinical studies, the antiphlogistic properties of comfrey extract could be demonstrated in various indications (Petersen *et al*., 1993; Koll *et al*., 2004; Predel *et al*., 2005; D'Anchise *et al*., 2007; Grube *et al*., 2007; Giannetti *et al*., 2010).

The efficacy of comfrey root extract ointment was evaluated in a randomised, double-blind, placebo-controlled multicentre study involving 142 patients with a unilateral ankle sprain. Compared to placebo, the superiority of the verum treatment was significant (Koll *et al*., 2004). The same ointment was compared with a gel preparation containing 1% of diclofenac in a randomised, single-blind multicentre study involving patients with the same condition. The results showed that the comfrey ointment was not inferior to diclofenac gel (Predel *et al*., 2005). In some variables there was even evidence of superiority of the comfrey ointment (D'Anchise *et al*., 2007).

Another randomised, double-blind, placebo-controlled clinical trial investigated the effect of the same ointment over a 3-week period in 220 patients with painful osteoarthritis of the knee. The superiority (*p* < 0.001) of the verum group over the placebo group was confirmed. Pain was reduced, mobility of the knee improved, and quality of life increased (Grube *et al*., 2007).

The effect of two concentrations of topical, comfrey-based botanical creams containing a blend of tannic acid and eucalyptus was compared to a eucalyptus reference cream in patients with primary osteoarthritis of the knee. Both active topical comfrey formulations were effective in relieving pain and stiffness and in improving physical functioning and were superior to placebo (Smith and Jacobson, 2011).

In the treatment of acute upper or low back pain, a study was conducted as a double-blind, multicentre, randomised clinical trial with parallel group design over a period of 5 days (Giannetti *et al*., 2010). One-hundred and twenty patients with acute upper or lower back pain were treated three times a day, 4 g per application. They used either a verum cream containing comfrey root fluid extract (1:2, 35.0 g, extraction solvent ethanol 60% (v/v), less than 0.35 ppm of pyrrolizidine alkaloids, Kytta-Salbe® f) or a corresponding placebo. The trial included four visits and was performed at the German Sport University in Cologne (Deutsche Sporthochschule) and three additional ambulatory centres for orthopaedics and sports medicine. The primary efficacy variable was the area under the curve (AUC) of the visual analogue scale (VAS) on active standardised movement values at visits 1 to 4. The pain intensity on VAS was assessed at performance of standardised, muscle group specific tests. The secondary objectives were back pain at rest using assessment by patient on VAS, pressure algometry (pain–time curve; AUC over 5 days), global assessment of efficacy by the patient and the investigator, intake of analgesic medication, and functional impairment measured with the Oswestry Disability Index.

The results were clear-cut and consistent across all primary and secondary efficacy variables. Comfrey root extract showed a remarkably potent, fast-acting, and clinically relevant effect in reducing acute back pain. The pain intensity on active standardised movement decreased on average (median) approximately 95.2% in the comfrey extract group (104.8–12.7 mm; mean VAS sum) and 37.8% in the placebo group (100.0–56.5 mm; mean VAS sum) (*p* < 0,001). Compared with placebo, superiority of the verum treatment was significant with regard to secondary efficacy variables (each *p* < 0.001). Both the AUC of the reported back pain at rest, the AUC of the pressure algometry in the trigger point, as well as the global assessment of the efficacy by the patients and the investigators showed a clinically relevant effect in reducing acute back pain. For the first time, a fast-acting effect of the ointment (1 h) was also observed. After 1 h, the pain intensity had already decreased about 33.0% in the comfrey group (104.8 to 60.4 mm; mean VAS sum) and 12.0% in the placebo group (100.00 to 86.5; mean VAS sum) indicating an early onset of the treatment effect.

Besides creams with the single comfrey root extract, a combination with methyl nicotinate has been used for decades. A non-published pilot study showed a favourable effect of the combination in patients with lumbar spine syndrome. Furthermore, the antiphlogistic and analgetic properties of the drug were evaluated in a post-marketing surveillance study. A total of 167 patients who used the preparation externally for contusions and distortions, muscle and joint pain were documented. The key symptoms were clearly reduced (Klingenburg, 2004).

It is therefore justified and reasonable to assume a beneficial effect of the combination of comfrey root extract and methyl nicotinate also in patients suffering from acute back pain such as upper or low back pain and to conduct a GCP-compliant clinical trial to document this assumed efficacy. For acute back pain, the Note for Guidance on Clinical Investigation of Medicinal Products for Treatment of Nociceptive Pain requires a study period of less than 1 week (Committee for Proprietary Medicinal Products, 2002).

## METHODS

The study was conducted at six active study centres in Germany. The Ethics Committee of the Ärztekammer Nordrhein, Düsseldorf, Germany, approved the protocol on 14 July 2009. We did not fully comply with the guideline available for the treatment of low back pain, as we included also patients with acute upper back pain into the trial (Devogelaer *et al*., 2003). However, this trial met most of the criteria mentioned for patients with types 1–2 low back pain.

### 

#### Participants

Main criteria for inclusion were: (i) Age range 18–45 years; (ii) Good general condition; (iii) Written informed consent; (iv) Acute back pain (either upper or low back pain), not in combination; (v) Sensitivity to algometric pressure on the site contralateral to the painful trigger point at least 2.5 N/cm²; (vi) Back pain on active standardised movement of at least 50 mm on a 100 mm VAS; (vii) Basic value of the pressure algometry on the trigger point should not exceed 50% of the respective value of the site contralateral to the painful trigger point.

Among the exclusion criteria were: (i) Upper or low back pain that was attributable to any identifiable cause (e.g. disc prolapse, spondylolisthesis, osteomalacia, or inflammatory arthritis); (ii) Any recent trauma; (iii) Any recent strains of the back muscles documented by the clinical evaluation and anamnesis; (iv) Chronic back pain; (v) Likelihood of prolapsed spinal disc documented by clinical symptoms (pain irradiation to peripheral areas, paraesthesia, clinically detectable impairment of muscle strength of related areas); (vi) Back pain caused by metabolic or neurological diseases documented by anamnesis (i.e. toxic neuropathy); (vii) Diabetes mellitus; (viii) Risk factors for spinal infection; (ix) Recent onset of bladder dysfunction or severe or progressive neurological deficit in the low extremity (as a possible indication of prolapsed disk); (x) Concomitant use of any anti-inflammatory drugs, heparinoids, or analgesics including herbal preparations (glucocorticosteroids, NSAIDs, etc.) for the same indication or other indications (e.g. rheumatoid arthritis); (xi) Analgesics or NSAIDs applied by any route of administration within 10 days before study entry or corticoid drugs applied by any route of administration within 60 days before study entry; (xii) Any other concomitant treatment (e.g. cosmetics, ointments on the treated area) or medication that interferes with the conduct of the trial. Patients with depression or other psychiatric disorders were not excluded. The distribution of those patients in the groups is stated in Table 1.

**Table 1:** Demographic, baseline, and other group characteristics (FAS/ITT)

			Combination	Methyl nicotinate	Placebo	Total
			
			(n = 163)	(n = 164)	(n = 52)	(n = 379)
Sex	male	n (%)	82 (50.3)	81 (49.4)	29 (55.8)	192 (50.7)
	female		81 (49.7)	83 (50.6)	23 (44.2)	187 (49.3)
Age (years)		Mean	31.29	29.02	28.92	29.98
		SD	8.48	8.31	7.70	8.36
Height (cm)		Mean	174.15	174.05	174.52	174.16
		SD	9.44	9.75	9.02	9.50
Weight (kg)		Mean	76.67	72.51	77.83	75.03
		SD	17.32	15.21	18.74	16.75
Ethnic origin	Caucasian	n (%)	163 (100)	163 (99.4)	50 (96.2)	376 (99.2)
	African		-	-	1 (1.9)	1 (0.3)
	Asian		-	1 (0.6)	-	1 (0.3)
	Other		-	-	1 (1.9)	1 (0.3)
Known allergies	yes	n (%)	43 (26.4)	34 (20.7)	9 (17.3)	86 (22.7)
	no		120 (73.6)	130 (79.3)	43 (82.7)	293 (77.3)
Concomitant medication	Antidepressants	n (%)	2 (1)	2 (1)	0	4 (1)
Concomitant diseases	Psychiatric disorders	n (%)	15 (9)	12 (7)	3 (5.8)	30 (7.9)
Mean daily dose of IMP		g	10.4	10.27	10.61	10.37
Localisation of back pain n (%)		upper	100 (61)	88 (54)	30 (58)	218 (56)
		low	63 (39)	76 (46)	22 (42)	161 (44)
Back pain on standardised movement at baseline, sum of VAS (mm)		Mean	164.57	157.73	165.38	-
		SD	43.62	40.87	43.74	-

#### Study design

The Phase III trial was conducted as a double-blind, multicentre, randomised, placebo-controlled clinical trial with three independent treatment groups (parallel group design). After obtaining written informed consent and after all patient eligibility reviews had been performed, patients were randomised and subsequently treated until Day 5 (fourth day after enrolment). One group of patients received a combination cream containing 35% of comfrey root extract (1:2, extractant 60 v/v%), and 1.2% methyl nicotinate (Kytta-Balsam® f, Merck Selbstmedikation GmbH, Darmstadt, Germany), the second group a cream containing 1.2% methyl nicotinate, and the third a placebo cream. Treatment was started after trial enrolment (visit 1). Patients were seen for evaluation of treatment effects after 1 h (visit 2) and after 3 and 5 days (± 1 day) (visits 3 and 4).

For scientific reasons, a parallel-group design was selected as the most suitable and generally accepted method. A double-blind treatment was chosen to avoid bias in the assessment of treatment success, and a randomisation was carried out to avoid a bias of treatment allocation. A placebo concurrent control was used because the aim of the study was to verify the combination's efficacy in patients with acute upper and low back pain. Placebo treatment was justified because the patients to be included in this clinical trial could ethically be treated with paracetamol as rescue medication. The study was to show that combination treatment is superior to single component. Therefore, a methyl nicotinate arm was included.

The randomisation ratio 3:3:1 was chosen because placebo was expected to be detectable by some of the patients due to the absence of the visible effects on the skin caused by methyl nicotinate. In order not to treat more patients than absolutely necessary only with placebo, and knowing the possibly limited feasibility of blinding the placebo arm, the placebo group was limited to the minimum number required to form a baseline.

This trial design was discussed and agreed in a scientific advisory procedure with the relevant German national authority, Bundesinstitut für Arzneimittel und Medizinprodukte (BfArM). Parties agreed to conduct this trial as a sibling study of the above mentioned back pain trial with comfrey root extract (Giannetti *et al*., 2010) and to use the most similar design possible. The full concept and the results that can be taken from both trials in combination will be subject to a seperate publication under preparation.

With respect to a homogeneous trial population, age range and a lack of clear diagnosis of acute upper or low back pain seemed to be the most important factors causing bias in baseline values. Therefore, only young patients (age 18–45 years) were included as postulated in the inclusion criteria. Moreover, only acute back pain cases were enrolled in order to exclude upper or low back pain that is attributable to any identifiable cause or trauma.

#### Interventions

A 12 cm long cream layer (corresponding to about 4 g) was spread onto the area of treatment and distributed by soft massage. A glove must be worn. The cream had to be administered three times a day in intervals of about 8 h and applied for 5 days. To ensure the patients applied the cream every time to the same area of the skin, the investigator marked the treatment area at the edges by means of a water-resistant pen.

In order to be able to control the applied amount of cream, patients were supplied with packs of gloves which were equipped with a printed measuring scale. The investigators as well as the patient information instructed the patients to squeeze out a continuous strand of cream to the hand wearing the glove and to compare it to the scale. Moreover, at each visit, the tube weight was measured with standardised weighing machines at the study centres. The tube weight was recorded in the CRF. Values for mean daily cream doses used by the patients are shown in Table 1.

Paracetamol tablets were allowed as rescue medication for breakthrough pain (maximum dose 4000 mg per 24 h; single dose: not more than 10–15 mg/kg at minimum intervals of 4–8 h) and had to be documented.

#### Outcome measures

##### Efficacy variables

The primary efficacy variable was the AUC of the VAS on active standardised movement values at visits 1 to 4 (at actual measurement times). For the calculation of the primary variable, the sum of the VAS for low back pain and for upper back pain was calculated using the actual measurement times, respectively. The AUC was determined using the cumulative trapezoidal rule. Higher values of the AUC of VAS sum on active standardised movement indicate higher pain.

The tests on active standardised movement were performed in a specific manner for each muscle (Lenhart and Seibert, 2001). For M. trapezius (upper part): The patient was sitting on a chair with the investigator standing behind him/her and fixing his/her shoulders. The patient pulled her/his head sideways towards the left of the right shoulder without lifting up the shoulder at the same time and reported the pain sensation. For M. latissimus dorsi, M. teres major: The patient was lying with the front on a table. The arm was adducted and rotated inwards. Palm was showing upward and prevents a rotation outwards. The patient tried to lift the arms upwards away from the table and reported the pain sensation. For M. deltoideus pars spinalis: The patient was lying with the front on a table, and the tested arm is abducted in a right angle with the forearm hanging over the edge of the table. The investigator fixed both the shoulder and the arm to the table surface with a gentle pressure. The patient tried to lift the arm upwards away from the table surface against the gentle resistance of the investigator's arms and reported the pain sensation. For M. erector spinae: The patient was lying with his/her front on a small table. The hips did not lie on the table. With her/his hands, the patient held on to the right and left edges of the table. The patient bended her/his legs to reach a square angle, lifted her/his bottom up towards the horizontal line, and reported the pain sensation. For M. rectus abdominis: The patient was lying on the back, legs extended, arms crossed behind his/her head. Both legs are stretched and lifted up to reach a right angle to the surface the patient is lying on. The spine did have full contact to the surface. The patient slowly lifted down to the surface the stretched legs and reported the pain sensation.

Secondary objectives of the study were the investigation of the following variables: Back pain at rest, assessment by patient on VAS, pressure algometry (pain–time curve; AUC over 5 days), Global assessment of efficacy by patient, Global assessment of efficacy by investigator, Functional impairment measured with the Oswestry Disability Index, consumption of analgesic medication. Moreover, the safety was assessed by means of general physical examinations, vital signs, and the occurrence of adverse events (AEs) and serious adverse events (SAEs), respectively.

#### Sample size

The sample size for this study was calculated based on the results of previous trials in particular involving patients with acute back pain. The sample size estimation was carried out by means of the software program Nquery and based on the assumption on a level of significance *α* = 5%, power 1-*β* = 80%, two-sided *t*-test situation, standardised difference (mean difference divided by standard deviation) *δ* = *Δ*/SD = 0.40 between the combination comfrey plus methyl nicotinate and single methyl nicotinate. At least 100 evaluable patients per treatment group had to be enrolled in the two active treatment groups. As the BfArM recommended to observe at least 300 patients under methyl nicotinate for safety reasons, it was decided to enrol a total of 350 patients (combination: *n* = 150, methyl nicotinate: *n* = 150, placebo: *n* = 50 using a 3:3:1 allocation ratio). However, a total of 378 patients had to be enrolled, because an approximate drop-out rate of 7% was expected. Each centre had to enrol at least 20 and at most 115 patients.

#### Criteria for evaluation

All randomised patients were assessed in the Full Analysis Set/intention-to-treat (FAS/ITT) population. Moreover, a per protocol analysis (PP) was performed. The PP population included all patients who met the inclusion and exclusion criteria and showed no major protocol violations. The FAS/ITT-evaluation was the primary analysis set in this superiority trial. All patients treated at least one time with one of the study drugs were assessed for safety.

## RESULTS

**Figure 1:**
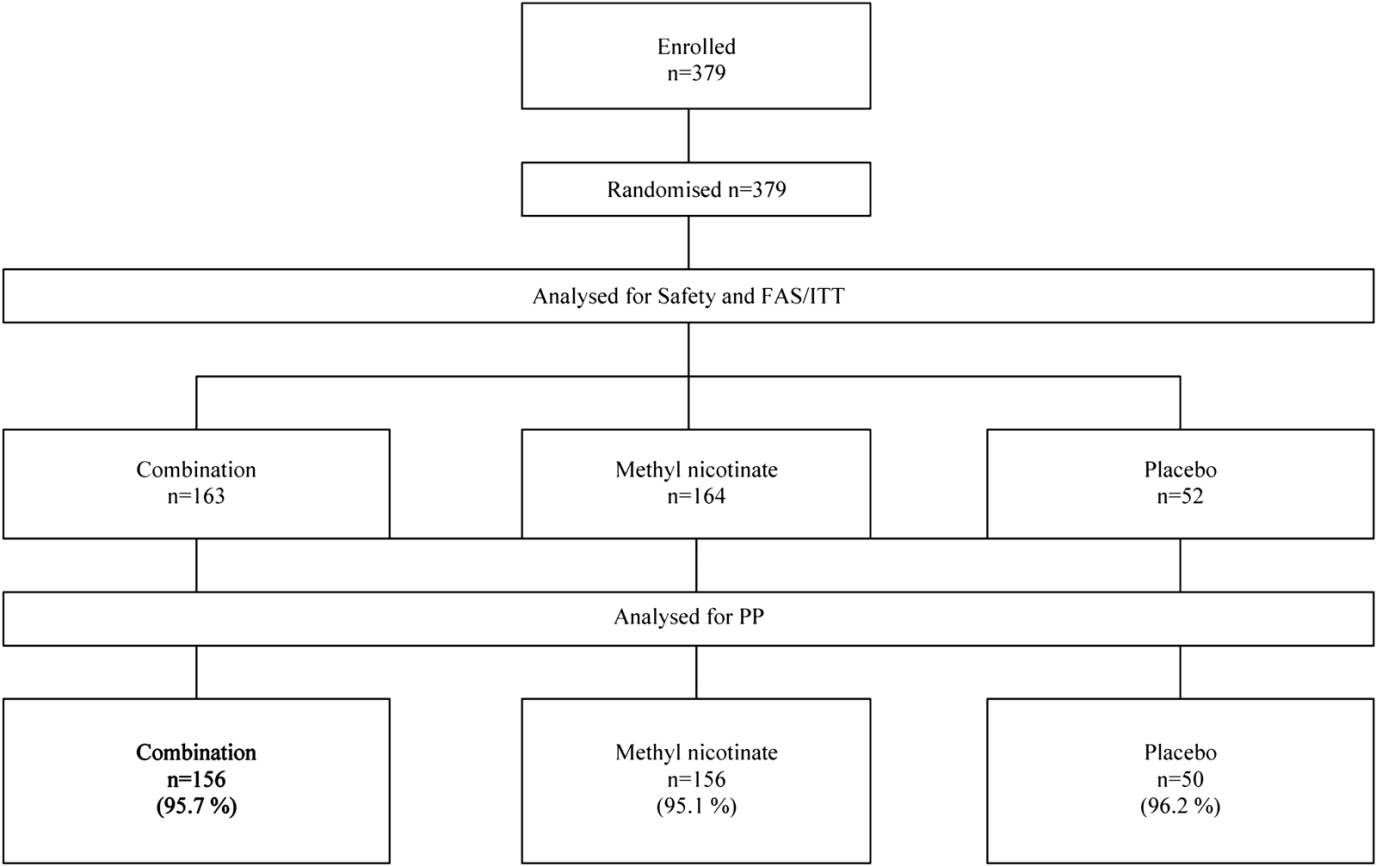
Patient's analysed flowchart.

Between January 2010 and May 2011, a total of 379 patients with conditions of acute upper or low back pain were randomly assigned to the double-blind treatment (combination: *n* = 163, methyl nicotinate: *n* = 164, placebo: *n* = 52). For efficacy, all enrolled patients were evaluated as the FAS/ITT population. After exclusion of 17 patients due to major protocol violations, a total of 362 patients (combination: *n* = 156, methyl nicotinate: *n* = 156, placebo: *n* = 50) were evaluated as PP population (Fig. 1). The treatment groups were well balanced with regard to the baseline characteristics (Table 1).

### Efficacy

#### Primary response criterion

The AUC of the VAS on active standardised movement values at visits 1 to 4 (at actual measurement times) was markedly smaller in the combination treatment group than in the methyl nicotinate and in the placebo group (ANOVA: *p* < 0.0001) (Table 2).

**Table 2:** AUC of VAS sum on active standardised movement at actual measurement times (FAS/ITT)

		Combination	Methyl nicotinate	Placebo
		
		(n = 163)	(n = 164)	(n = 52)
AUC – sum of VAS values at actual measurement times	Mean	6548.65	8975.32	13052.40
(mm x h)	SD	4021.33	3635.20	4567.87
	Median	6013.56	8769.48	13223.31
p (ANOVA)			<.0001	

**Figure 2:**
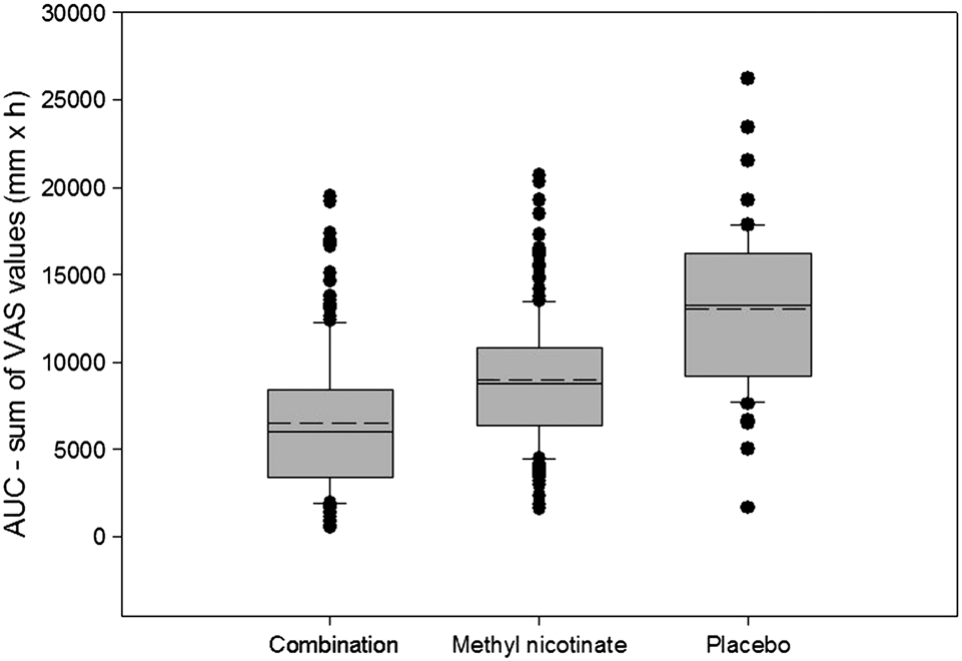
AUC of VAS sum on active standardised movement at actual measurement times (FAS/ITT).

The pairwise comparisons of the mean AUCs of VAS sums on active standardised movement showed values 27% lower in favour of the combination compared to methyl nicotinate (6548.65 mm × h versus 8975.32 mm × h, i.e. a mean treatment effect of −2426.7 mm × h), and values 50% lower in favour of the combination compared to placebo (6548.65 mm × h versus 13052.40 mm × h, mean treatment effect −6503.8 mm × h). Methyl nicotinate alone reached a reduction in this variable of 31% compared to placebo (8975.32 mm × h versus 13052.40 mm × h, mean treatment effect 4077.1 mm × h). All pairwise comparisons were statistically significant (*t*-test: *p* < 0.0001). The combination proved superiority to the two other treatment arms (Fig. 2).

#### Secondary response criteria

The pairwise comparisons of the AUC of VAS values on pain at rest at actual measurement times were 27% lower, comparing the combination with methyl nicotinate (1782.60 mm × h versus 2457.32 mm × h, mean treatment effect −674.7 mm × h), and 54% lower comparing the combination to placebo (1782.60 mm × h versus 3910.66 mm × h, mean treatment effect −2128.1 mm × h); all pairwise comparisons were again statistically significant (*t*-test: *p* = 0.0005, *p* < 0.0001). The AUC of the pressure algometry values in the trigger point was much higher in the combination group, which means significantly less inducible pressure pain than in both comparator groups (64% more compared to placebo and 19% more compared to methyl nicotinate, respectively, *t*-test: *p* < 0.0001).

**Figure 3:**
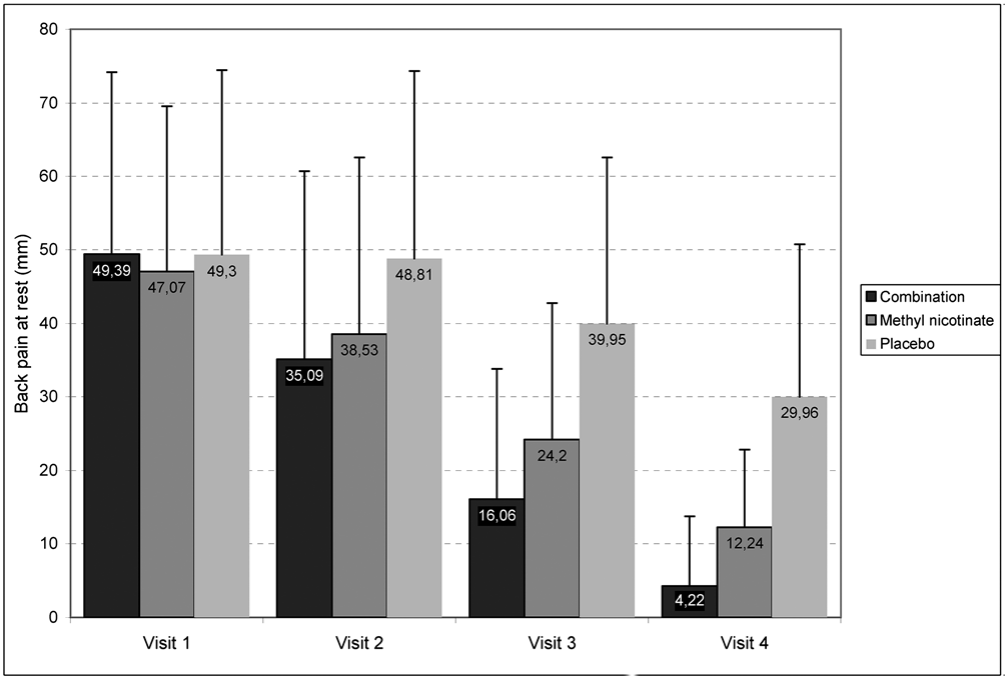
VAS pain at rest – means and SD (FAS/ITT).

Similar to the mean reduction of pain on movement values, the mean pain-at-rest values – assessed also by using VAS – decreased statistically significant (ANOVA: *p* < 0.0001) in the actively treated groups compared to placebo (Fig. 3). The pairwise comparisons of the AUC of VAS pain at rest at actual times were −674.7 mm × h in favour of the active combination compared to methyl nicotinate and nearly the triple in comparison to placebo (−2128.1 mm × h).

The VAS sum of pain on active standardised movement diminished after 1 h (visit 2) by 25.2% in the combination group, by 15% in the methyl nicotinate group, and by 4,1% in the placebo group. The VAS sum of pain at rest reduced at the same visit 2 by 29% in the combination group, by 18.1% in the methyl nicotinate group, but only by 1% in the placebo group.

The global assessments of efficacy by investigators and patients showed that the difference between the treatment groups was statistically significant at visit 4 (CMH: *p* < 0.0001). The percentage of patients who were assessed as ‘good’ or ‘excellent’ in terms of global efficacy was markedly higher in the combination group (93.3%) compared to methyl nicotinate (51.2%) and placebo (7.6%). Moreover, ‘no’ or ‘poor’ global efficacy was rarely documented in actively treated patients, in contrast to placebo. The assessment of global efficacy coming from the patients was very similar. In the comfrey group, the rating of ‘good’ or “excellent” was 93.9%, compared to methyl nicotinate (49.4%) and placebo (7.7%).

Moreover, 17.3% of patients in the placebo group needed to take paracetamol at least once as rescue medication, which was markedly more than in the treatment groups (combination group: 9.2%, methyl nicotinate group: 5.5%). The average total dose of rescue medication was higher in the placebo group (9929 mg) than in the active treatment groups (combination: 7400 mg, methyl nicotinate: 8889 mg) in the subgroup of patients who took rescue medications at least once.

The Oswestry Disability Index between visit 1 and visit 4 (FAS/ITT) improved by 80% in the combination group (V1 24.85, V4 4.96) compared to 54% in the methyl nicotinate group (V1 24.38, V4 11.3) and 22% in the placebo group (V1 25.63, V4 20.06).

The combination ointment was thus consistently more effective in the treatment of acute upper or low back pain than both comparators, while methyl nicotinate displayed a non-negligible effect as well. The results are consistent across the primary and all secondary variables in this clinical trial. Patients treated with the combination had significant reductions in pain scores and were more satisfied with the treatment effect than those receiving only methyl nicotinate. A clear benefit of the combination product compared to the mono substance methyl nicotinate could thus be proven. Methyl nicotinate alone, however, proved to have a noticeable effect on the disease under investigation as well.

### Safety

All 379 patients enrolled, who received at least one dose of study medication, were included in the safety population (SAF). A total of 327 patients received at least one dose of one of the nicotinate-containing preparations (combination: *n* = 163, methyl nicotinate: *n* = 164). Fifty-two patients received placebo. AEs reports, which included multiple descriptions, were split into single AEs for analysis purposes and counted separately. Four drug-related AEs were recurrent, meaning they were recorded more than once in the same patient.

A total of 19 patients (5%) (combination: *n* = 10 (6.1%), methyl nicotinate: *n* = 9 (5.5%), placebo: *n* = 0) showed at least one AE during the course of the clinical trial (33 AEs in total). 9 patients (2.4%) showed at least one AE, which was classified as drug-related (combination: *n* = 3 (1.8%), methyl nicotinate: *n* = 6 (3.7%), placebo: *n* = 0). In total, 22 drug-related AEs were recorded.

The System Organ Class which was affected most frequently was ‘General disorders and administration site conditions’. All drug-related events were application site reactions (application site erythema, hypersensitivity, pruritus, and reaction); they represent typical reactions caused by the topical application form of the treatments. It is remarkable that the majority of AEs out of these drug-related cutaneous side effects (88%) occurred in the methyl nicotinate group (15 application site reactions), not in the combination group (two application site reactions).

The 22 drug-related AEs (certain or probable) also included recurrent events, and all occurred in the two treatment groups, none in the placebo-group (combination: *n* = 7, methyl nicotinate: *n* = 15). The causality of the seven drug-related AEs in the combination group was assessed as ‘probably’ drug related. The 15 drug-related AEs in the methyl nicotinate group were classified as ‘certainly’ drug-related. All above drug-related AEs were assessed as mild or moderate by the investigators.

Two unrelated AEs were classified as serious: recurrent depressive disorder and pancreatic insufficiency. Both of these cases occurred in the combination treatment group.

## DISCUSSION

The results demonstrate that the topical combination of comfrey root extract and methyl nicotinate has a clinically relevant, favourable impact on the outcomes of patients suffering from acute upper or low back pain. Patients treated with the combination had statistically significant and clinically relevant reductions in pain scores and increases in tenderness. Significantly more patients in the combination group reached a virtually pain-free status at visit 4 compared to the placebo group as well as compared to the methyl nicotinate group, as documented by the results of the global assessment of efficacy by the patients and by the investigators. They reached a pain-free condition significantly earlier than patients in the methyl nicotinate and those in the placebo group.

Further, the clinical trial showed that methyl nicotinate contributes substantially to the efficacy of the combination product, reducing the primary parameter by 31% compared to placebo.

Patients using topical drugs for self-medication do not usually know the precise cause of their pain; however they do seek pain relief. This trial might have limitations as we did not include, for example, a pain diary, which may have allowed further, relevant, detailed data analysis. However, the efficacy demonstrated reflects the pharmaceutical treatment routine of this patient group to quite an extent.

## CONCLUSION

The combination of comfrey root extract plus methyl nicotinate was consistently more effective in the treatment of acute upper or low back pain than both comparators, while methyl nicotinate displayed an effect as well. The clinical trial at hand confirms the topical combination is an effective and well-tolerated treatment option for acute back pain.

## CONTRIBUTORSHIP STATEMENT

Helmut Pabst and Axel Schaefer were investigators, Hans-Georg Predel was principal investigator of this RCT, Marc Junker-Samek was project manager, Christiane Staiger was project manager and wrote most of the article.

## FUNDING

Merck Selbstmedikation GmbH sponsored the study. The study was conducted by the CRO CRM clinical trials GmbH, Rheinbach, Germany.
